# Nanophotonics of higher-plant photosynthetic membranes

**DOI:** 10.1038/s41377-018-0116-8

**Published:** 2019-01-09

**Authors:** A. Capretti, A. K. Ringsmuth, J. F. van Velzen, A. Rosnik, R. Croce, T. Gregorkiewicz

**Affiliations:** 1 Institute of Physics, University of Amsterdam, Amsterdam, Netherlands; 2 Dep. Physics and Astronomy, VU University Amsterdam, Amsterdam, Netherlands; 3 College of Chemistry, University of California, Berkeley, CA USA; 4 Present Address: Stockholm Resilience Centre, Stockholm University, Stockholm, Sweden

**Keywords:** Biophotonics, Nanophotonics and plasmonics

## Abstract

The thylakoid membrane inside chloroplasts hosts the light-dependent reactions of photosynthesis. Its embedded protein complexes are responsible for light harvesting, excitation energy transfer, charge separation, and transport. In higher plants, when the illumination conditions vary, the membrane adapts its composition and nanoscale morphology, which is characterized by appressed and non-appressed regions known as grana and stroma lamellae, respectively. Here we investigate the nanophotonic regime of light propagation in chloroplasts of higher plants and identify novel mechanisms in the optical response of the thylakoid membrane. Our results indicate that the relative contributions of light scattering and absorption to the overall optical response of grana strongly depend on the concentration of the light-harvesting complexes. For the pigment concentrations typically found in chloroplasts, the two mechanisms have comparable strengths, and their relative value can be tuned by variations in the protein composition or in the granal diameter. Furthermore, we find that collective modes in ensembles of grana significantly increase light absorption at selected wavelengths, even in the presence of moderate biological disorder. Small variations in the granal separation or a large disorder can dismantle this collective response. We propose that chloroplasts use this mechanism as a strategy against dangerously high illumination conditions, triggering a transition to low-absorbing states. We conclude that the morphological separation of the thylakoid membrane in higher plants supports strong nanophotonic effects, which may be used by chloroplasts to regulate light absorption. This adaptive self-organization capability is of interest as a model for novel bioinspired optical materials for artificial photosynthesis, imaging, and sensing.

## Biophotonics: Shape-shifting discs help plants avoid overexposure

The ability of plants to survive dangerous illumination conditions may be related to photonic structures inside light-harvesting membranes. Antonio Capretti from the University of Amsterdam in the Netherlands and colleagues used computer simulations to study thylakoid membranes in plant cells, thin disc-shaped structures that are the active site of photosynthetic reactions. They focused on the membrane’s nanoscale architecture by modeling the stacks of thylakoids, known as grana, which are known to adapt light-dependent conformations. Simulations of refractive index showed that the grana interacted collectively to enhance solar energy harvesting. However, small changes in the morphology of the stacks and the protein composition inside each thylakoid disc could trigger formation of a new photonic state that scatters incoming light—findings that may aid efforts to bioengineer photosynthetic organisms, such as microalgae.

## Introduction

**Fig. 1:**
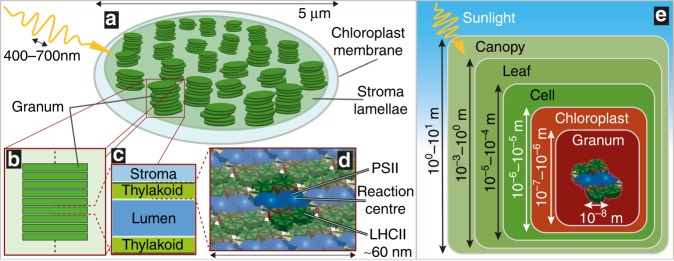
**a** Schematic of a higher-plant chloroplast showing the outer chloroplast membrane and the thylakoid membrane within. The thylakoid is morphologically separated into tightly stacked, discoidal grana, and unstacked stroma lamellae. The photosynthetically active wavelengths of the incident light are shown approximately to scale, corresponding closely to the sizes of grana while being two orders of magnitude larger than the embedded LHCs. **b** An individual granum schematized as a periodic stack of discoidal layers. **c** Each granal layer consists of four strata: two protein-embedding lipid bilayers (green), a lumen region (mid blue), and a thinner stroma region (light blue). **d** Schematic of an array of PSII-LHCII supercomplexes embedded in the lipid bilayer of the thylakoid membrane. **e** A picture of photosynthetic light harvesting in which the optical properties at each structural level provide an effective light environment for the next smaller level. Whereas the light distribution at higher levels (green) inhabits the ray optics regime, and excitation energy transfer within the protein complexes requires near-field quantum dynamical methods, the intermediate levels inside the chloroplast (red) demand a nanophotonic description

Photosynthesis, the conversion of sunlight into chemical energy, holds much promise for sustainable food and fuel production^1^. In higher plants, the light-dependent reactions of photosynthesis occur at the level of the thylakoid membrane inside chloroplasts. Under most physiological conditions, the thylakoid membrane of plants is morphologically separated into tightly stacked *grana* and non-appressed *stroma lamellae* connecting the grana (Fig. 1a, b)^2^. The grana are discoidal in shape, with diameters typically reported in the range from 200 to 600 nm^2,3^. The thylakoid membrane separates the chloroplast into two fluid spaces, known as the stroma and lumen (Fig. 1c). Although there are well-known photochemical benefits of this morphology^4,5^, the reasons for its evolution in higher plants remain only partially understood.

Embedded in the membrane, a variety of pigment–protein complexes are responsible for light harvesting, excitation energy transfer, and charge separation^6^. In particular, photosystems I and II (PSI and PSII) host the reaction centers (RCs), where charge separation occurs, and are surrounded by antenna complexes consisting of members of the light-harvesting complex (LHC) multigenic family that increase the absorption cross-section of the photosystems, harvest light, and transfer the excitation energy to the RCs (Fig. 1d)^2^. The protein distribution is highly heterogeneous, with PSI present in the stroma lamellae and PSII mainly present in the grana. LHCII, the main antenna complex of plants, is associated with both PSs and its amount varies depending on the light conditions, regulating the absorption cross-sections of the two PSs to balance their charge separation kinetics under the prevailing light conditions^7–10^. Owing to the different absorption properties of PSI and PSII, changes in the spectrum of the incident light generate an excitation imbalance. In reversible processes known at state transitions, a fraction of the LHCII complexes migrate between PSII and PSI complexes to rebalance their charge separation kinetics^11,12^.

Large differences are visible in the organization of the thylakoid membrane in plants that have adapted to different light conditions. The number of layers per granum is far larger in shade-adapted plants than in sun-adapted ones, while the number of grana per chloroplast is lower^8,13,14^. Under high light illumination, the lumen phase has been reported to reversibly expand, leading to swelling of the grana^4,15,16^. The granal diameter in spinach strongly decreases when the illumination conditions are changed from dark to light and increases again for very high light conditions^14^. Partial destacking with a reduction in the granal diameter has also been observed^17–19^. The electrostatic interactions between LHCIIs in adjacent membrane layers are responsible for stabilizing granal stacking, and LHCII phosphorylation during state transitions drives changes in the thylakoid morphology^2,20–22^.

It is important to point out that the structure, composition, and adaptation of thylakoid membranes vary significantly between species. Cyanobacteria are considered to be the evolutionary progenitors of the chloroplasts in green algae and plants^23^, but their thylakoids lack stacked granal regions. The photosystems in cyanobacteria differ in their protein organization compared with plants and show no obvious lateral heterogeneity^24^. In contrast to plants, their principal LHCs (called phycobilisomes) are large pigment–protein assemblies that are not embedded in the membrane but bind to the surface of the photosystems^25^. State transitions also regulate the energy balance between PSI and PSII in cyanobacteria, but their mechanisms are less understood than those in higher plants^24,26^. Green algal chloroplasts are more strongly evolutionarily linked to chloroplasts in higher plants, with similar LHCs embedded in the membrane^27^. Their thylakoids are stacked but lack a clear segregation into grana and stroma lamellae^28^. Additionally, their state transitions use mechanisms closely analogous to those in higher plants^11^. In the present study, we focus on higher-plant thylakoids with known pigment concentrations and structures clearly segregated into grana and stroma lamellae.

A common picture of photosynthetic light harvesting shows sunlight incident directly on a pigment within a photosynthetic protein. A more complete picture accounts for the optical effects of large-scale structures, which mediate the interaction between the incident light and proteins (Fig. 1e). At each scale, plants have developed mechanisms to regulate light absorption, including leaf movements in the canopy, chloroplast movements in the cell^29,30^, and changes in the concentration and composition of the pigment–protein complexes at the molecular level^10^. Interestingly, the thylakoid ultrastructure, which is the intermediate level between cells and molecules, has received little attention despite the large variations mentioned above. While light transfer is well described by ray optics at the canopy, leaf, and cell levels, at the thylakoid level, the size and separation of the grana are comparable to the wavelengths of photosynthetically active radiation (400–700 nm by convention), and a wave description is required. Nanophotonic effects arising in this regime are known to lead to counterintuitive optical effects in dielectric nanoparticles, including directional scattering, light trapping, and enhancement, focusing below the diffraction limit and Mie resonances^3,31–33^.

In plants, photonic effects are mostly known to give rise to structural color^34^. Preliminary numerical studies have highlighted the importance of diffraction effects and the unsuitability of ray optics at the thylakoid level^32^. A novel study showed that iridoplasts in the shade-dwelling species of Begonia feature thylakoid membranes organized in a planar multilayered structure, giving rise to photonic crystal properties^33^. Additionally, it was explicitly proposed that the inner structure of chloroplasts has photonic functionalities in addition to the established photochemical ones. Another recent work utilized elastic light scattering to assess the structure of thylakoid membranes^3^. The work experimentally showed that grana are efficient nanoscatterers of visible light and are biological analogs of dielectric nanoparticles. These converging lines of evidence suggest that the morphological separation of the thylakoid membrane facilitates significant nanophotonic effects. There is now an urgent need to better understand these effects to help decipher the multiple functionalities of the thylakoid membrane and develop strategies for improved photosynthetic performance.

In the present work, we address the nanophotonic functionalities of the thylakoid membrane in higher plants with rigorous full-wave computational methods, developing models of increasing complexity and taking into account the morphological disorder observed in structural studies of chloroplasts. We find that nanophotonic effects help to regulate light absorption in the thylakoid membrane. While it is well established that the thylakoid’s complex morphology and composition have multiple photochemical functions, our results provide new insight into their capacity for light management through nanophotonic mechanisms. These results apply most directly to higher-plant chloroplasts, but our methods can also be utilized for the thylakoids of other species with known structure and pigment composition. We, therefore, open up a powerful, novel approach for studying the interplay between thylakoid composition, structure, and nanophotonic effects. In particular, the adaptive self-organization capabilities of the thylakoid membrane are of great interest for developing novel bioinspired optical materials with applications that include energy, imaging, and sensing. In future, we envision the possibility of engineering adaptive, self-organizing light-harvesting systems for maximally productive artificial photosynthesis to help meet the growing demands for energy.

## Results

### Preliminaries

We model a granum as a stack of discoidal layers, with each made of four strata, as shown in Fig. 1b, c: the two strata of the thylakoid membrane, the lumen, and the stroma (with thicknesses *t*_thylakoid_, *t*_lumen_, and *t*_stroma_, respectively). In this simple schematic, we neglect the lipid bilayer regions connecting adjacent layers (the so-called granal margins) and any edge features, such as the lamellae departing from the granum. The optical response of each constituent is characterized by the refractive index. Because direct experimental determination is not available for such nanoscale objects^35^, we utilize effective medium approximations, since the individual strata have thicknesses (~≤10 nm) much smaller than the wavelengths of visible light. The thylakoid membrane mainly consists of a minority phase of galactolipids and a majority phase of functional proteins (with up to *f*_protein_ ~ 70–80% volume fraction)^36^. Both phases are characterized by their own refractive index; therefore, the thylakoid is a heterogeneous medium with intermediate properties between the two phases. We calculate its effective index *ñ*_thylakoid_ = *n*_thylakoid_ + *i·k*_thylakoid_ (where the tilde indicates a complex value) by using Brugmann’s theory, as explained in Materials and methods. Both chlorophylls and carotenoids, mostly contained in the protein phase, determine the extinction coefficient *k*_thylakoid_, which takes into account the absorption of light. Notably, chlorophylls dominate at the wavelength of interest, *λ* = 680 nm, which corresponds to the spectral peak of the RC P680 of PSII.

**Fig. 2:**
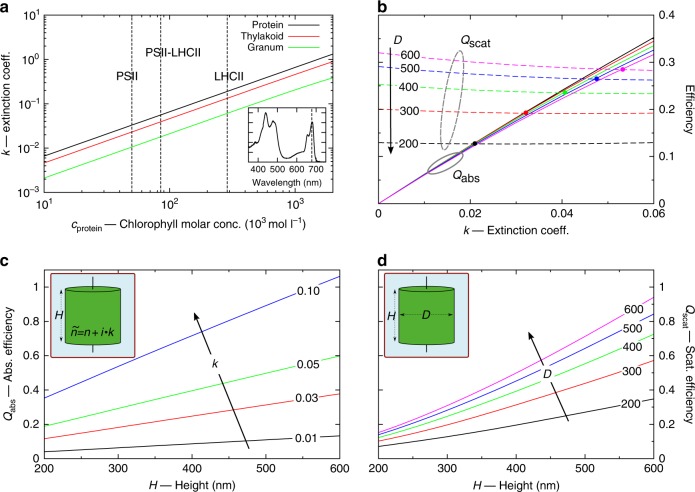
**a** Extinction coefficient of the protein phase (black), the thylakoid stratum (red), and the granal layer (green) as a function of the chlorophyll molar concentration *c*_protein_ (with respect to the protein phase) at *λ* = 680 nm. The inset shows a representative absorption spectrum of LHCIIs. **b** Absorption (continuous lines) and scattering (dashed lines) efficiencies of cylindrical grana as a function of the extinction coefficient *k* of the granal layer. The granal height is *H* = 300 nm, and the diameter *D* is parametrized from 200 to 600 nm. **c** Absorption efficiency of cylindrical grana with *D* = 300 nm as a function of *H*, parametrized for *k* from 0.01 to 0.1. **d** Scattering efficiency of cylindrical grana with *k* = 0.01 as a function of *H*, parametrized for *D* from 200 nm to 600 nm. All calculations are at *λ* = 680 nm

We then attribute a homogeneous isotropic effective refractive index *ñ* = *n* + *i·k* to the discoidal layers comprising the grana using the theory of periodic stratified media (see Materials and methods). The difference in the real part of the refractive indices between the aqueous and the thylakoid strata is relatively small (<15%). Therefore, variations on the order of a few nm in the stratum thicknesses have limited effects on *n*. For all calculations presented, we set *n* = 1.55 (in agreement with previous literature) to maintain consistency and derive general properties (see also Materials and methods)^37^. On the other hand, the extinction coefficient *k* strongly depends on the chlorophyll concentration. Figure 2a shows the extinction coefficients at *λ* = 680 nm of the protein phase (black), thylakoid stratum (red), and granal layers (*k*, green) as a function of the chlorophyll concentration *c*_protein_ in the range from 10^4^ to 10^6^ mol L^−1^. The dependence, shown on a log–log scale, is nonlinear. For consistency, throughout this paper, the chlorophyll concentration is indicated with respect to the protein phase. Since there are neither PSIIs nor LHCIIs in the lipid and aqueous phases, the chlorophyll concentrations in the thylakoid membrane *c*_thylakoid_ and the granal layers *c*_layer_ are lower. Their values can be simply calculated by multiplication with the volume fractions *f*_protein_ and *f*_thylakoid_. For instance, the extinction coefficient *k* = 0.01 (green curve in Fig. 2a) corresponds to chlorophyll concentrations of *c*_protein_ = 60 mol L^−1^, *c*_thylakoid_ = 42 mol L^−1^, and *c*_layer_ = 16 mol L^−1^ if we assume *f*_protein_ = 70% and *f*_thylakoid_ = 39% (the latter value is obtained by assuming *t*_thylakoid_ = 3 nm, *t*_lumen_ = 7.5 nm, and *t*_*stroma*_ = 1.8 nm^16^). The chlorophyll concentration significantly varies with protein composition in the thylakoid membrane. Here we identify three reference cases of composition: (A) only LHCIIs, (B) both PSIIs and LHCIIs (in a 1:1 ratio), and (C) only PSIIs (see Supplementary Table S2). The vertical dashed lines in Fig. 2a mark these cases and help to identify the range from 0.01 to 0.05 as reasonable values for *k* (corresponding to *c*_protein_ = 60 mol L^−1^ and *c*_protein_ = 300 mol L^−1^, respectively).

### Light absorption and scattering by grana

Our study begins with the question of whether individual grana provide nanophotonic functionality in photosynthetic light harvesting, and we model the grana initially as homogeneous cylinders with height *H*, diameter *D*, and refractive index *ñ*. The incident light is a linearly polarized plane wave that propagates parallel to the granal axis, with wavelength *λ* = 680 nm. Figure 2b shows the absorption *Q*_abs_ and scattering *Q*_scat_ efficiencies of a granum as a function of *k* (for definitions, see Suppl. Info.), where *H* = 300 nm and *D* varies between 200 and 600 nm. For a fixed size, the scattering is approximately constant, whereas the absorption grows linearly with *k*; as a result, the two curves cross at a certain value of *k* (indicated by the dots). For instance, in the case with *D* = 200 nm, *Q*_abs_ = *Q*_scat_ at *k* ~ 0.02, corresponding to *c*_protein_ = 120 mol L^−1^. For lower values of *k*, the absorption decreases, whereas for *k* > 0.05, it becomes dominant (larger than twice the scattering). We conclude that there are two different optical states, depending on the granal size and chlorophyll concentration: one state is mostly scattering, where absorption is negligible, and one state is highly absorptive, where the absorption is a multifold of the scattering. For typical granal sizes and reasonable values of *c*_protein_, the scattering and absorption have comparable magnitudes, and this balance shifts with small variations in size and concentration. Figure 2b also shows that *Q*_abs_ is approximately independent of *D* (because the plane wave is perpendicular to the granal base), whereas Fig. 2c shows that *Q*_abs_ increases with *H*. This trend is similar to the Beer–Lambert law for a slab in the ray optics regime, and the trend is preserved for any value of *k*. Resonance modes are not observed, despite the granal size being comparable to the incident wavelength. This is due to the small difference between the refractive indices of the grana and surrounding stroma. Conversely, *Q*_scat_ increases with both *D* and *H*, as shown in Fig. 2d. For this reason, the value of *k* (and of *c*_protein_) at which *Q*_abs_ = *Q*_scat_ depends on the granal size.

These trends in the granal size are interesting in view of the experimental evidence that the diameter varies in thylakoids adapted to different light conditions, from dark to high light illumination^14^. Moreover, our results indicate that the chlorophyll concentration also affects the relative magnitudes of absorption and scattering. To quantify a reasonable range of variation in the relative magnitude, we consider two extremes: for high pigment concentrations and small granal size, *Q*_abs_ is a multiple of *Q*_scat_ (*Q*_abs_ ∼ 3·*Q*_scat_ at *c*_protein_ ~ 300 mol L^−1^ for *D* = 200 nm), while for low concentrations and large size, *Q*_scat_ is higher than *Q*_abs_ (*Q*_scat_ ∼ 6·*Q*_abs_ at *c*_protein_ ~ 60 mol L^−1^ for *D* = 600 nm). In the latter case, the grana redirect most of the incident radiation elsewhere. Inside chloroplasts, grana are found in closely spaced ensembles; therefore, a significant amount of light is scattered toward neighboring grana. Additionally, grana in proximity to the chloroplast envelope membranes redirect part of the incident radiation to neighboring chloroplasts. This might assist photoprotection and help to preserve the photosynthetic efficiency under high light conditions. Above the light absorption rate that saturates the photochemical kinetics, the photosynthetic efficiency declines as absorption increases, primarily due to the nonphotochemical quenching (NPQ) of excitons in the light-harvesting antennas. The reduced expression of LHCs under high light conditions results in a transition from the high-absorption regime to the high-scattering regime^38^, helping to reduce NPQ in the grana while also redistributing light to other chloroplasts where it may be used productively. We suggest that this mechanism may be a nanophotonic analog of the light-adaptive movements of chloroplasts and leaves at larger scales.

**Fig. 3:**
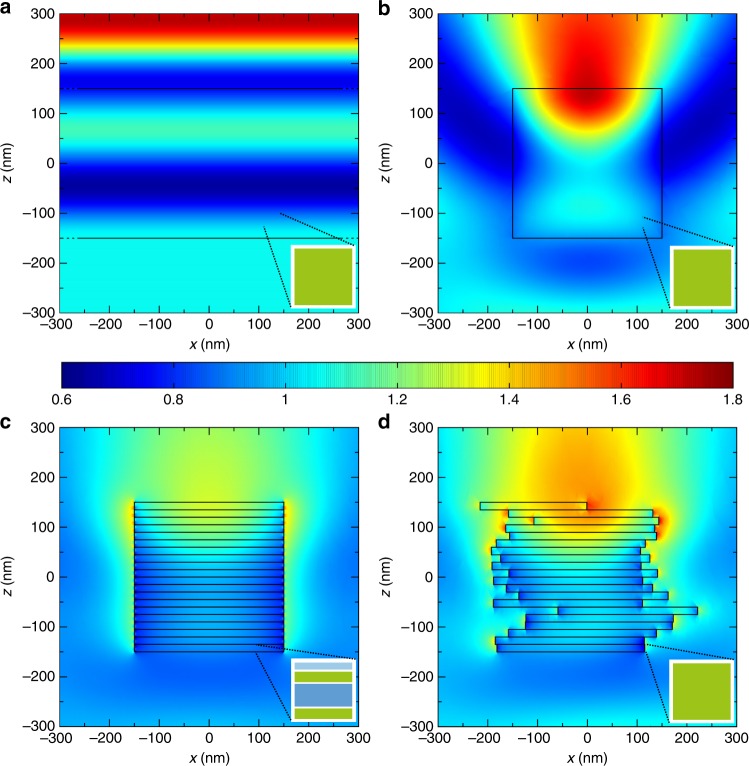
Spatial distributions of the light intensity in granal models of increasing complexity. **a** Reference case of a homogeneous slab with a 300 nm height, infinite width, and *k* = 0.01. **b** A cylindrical homogeneous granum with *H* = 300 nm, *D* = 300 nm, and *k* = 0.01. **c** An ordered granum made of 20 discoidal layers, each consisting of four strata with thicknesses of *t*_thylakoid_ = 3 nm, *t*_lumen_ = 7.5 nm, and *t*_stroma_ = 1.8 nm (the total granal size is approximately the same as in **b**). **d** A granum made of laterally displaced discoidal layers 15.3 nm in thickness. Each layer is homogeneous with *k* = 0.01. The lateral displacements with respect to the *x* and *y* axes are sampled from Gaussian distributions with *σ*_layer_ = 40 nm. The incident light is a plane wave propagating along the *z* axis from above with *λ* = 680 nm that is linearly polarized along the *x* axis with unitary intensity

The distribution of light absorption across a granal volume has important implications for its photosynthetic efficiency. Here we investigate the effect of granal size by calculating the spatial distribution of light across its volume, assuming normal incidence (along the *z* axis) from above and polarization along the *x* axis. It is important to note that the effective medium methods utilized for determining the refractive index imply a homogeneous distribution of absorbing chlorophylls across the whole granum. Figure 3a shows a reference case of an infinite slab, which has a finite height (300 nm) and lateral translational symmetry. The light intensity distribution shows the same symmetry, and inside the slab, it is simply determined by the interference between the incident and reflected waves. On the other hand, in a cylindrical granum with a homogeneous internal structure (*H* = 300 nm, *D* = 300 nm, and *k* = 0.01), the translational symmetry is removed because of its lateral walls. Consequently, light impinging on the granum scatters at its edges, and the intensity distribution loses symmetry, as shown in Fig. 3b. Notably, the intensity is maximal close to the top interface. Real grana comprise layers with internally stratified structures (Fig. 1b, c). Figure 3c shows the intensity for a periodic, layered morphology that also includes the internal strata of each layer: we fix the thicknesses *t*_thylakoid_, *t*_lumen_, and *t*_stroma_ at the values listed in Preliminaries, which sum to 15.3 nm, the total thickness of a granal discoidal layer. Each stratum has a different refractive index, as discussed in Preliminaries and Materials and methods. We find that the hot spot near the top interface is reduced by 20–30% with respect to the case of a homogeneous (nonlayered) granum of the same size (Fig. 3b). This indicates that reflection and scattering within the stratified structure facilitate a more homogeneous distribution of light absorption across the granum. Therefore, the stratified structure can help to increase the photosynthetic efficiency of the overall granum by reducing light supersaturation in some regions (and thus NPQ), while increasing productivity in regions that would otherwise be light limited.

**Fig. 4:**
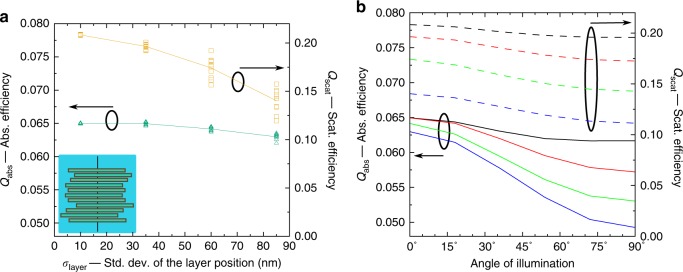
**a** Absorption (left axis, green) and scattering (right axis, yellow) efficiencies for a granum with laterally displaced discoidal layers as a function of the standard deviation of the position *σ*_layer_. The illumination source is a plane wave propagating parallel to the granal axis. **b** Absorption (left axis, continuous lines) and scattering (right axis, dashed lines) efficiencies as a function of the illumination angle *θ* of the plane wave for *σ*_layer_ = 10 nm (black), 35 nm (red), 60 nm (green), and 85 nm (blue). For each *σ*_layer_ and *θ*, the efficiencies are calculated as the average of ten different realizations of grana. All data are for *D* = *H* = 300 nm, *k* = 0.01 (*c*_protein_ = 60 mol L^−1^), and *λ* = 680 nm

We recall that structural studies have shown that the layers of plant grana are often misaligned and vary in width^2^. Our results in Fig. 3d show that the distribution of light intensity is approximately preserved in the presence of lateral displacements of the granal discoidal layers, featuring again an intensity maximum at the illuminated surface. The lateral displacements of the layers with respect to the *x* and *y* axes are sampled from Gaussian distributions with standard deviation *σ*_layer_ = 40 nm. We also see the appearance of additional small hot spots near some edges, which is a well-known phenomenon occurring in proximity to nanoscale dielectric tips. In Fig. 3d, the four-stratum structure used for each layer in Fig. 3c was again homogenized to reduce computational cost. The total thickness of the discoidal layers is the same for both panels (15.3 nm). It is reasonable to expect that restoring the internal stratification to the layers may again homogenize the intensity distribution. Supplementary Figure S3a shows the distribution of light intensity, calculated as an average of seven different realizations of grana with lateral displacements of their layers. This distribution strongly resembles the one for the cylindrical granum of Fig. 3b. Their relative difference is plotted in Suppl. Fig. S3b. The effects of the lateral displacement of the granal layers on *Q*_abs_ and *Q*_scat_ are shown in Fig. 4a: both efficiencies decrease as *σ*_layer_ increases. In the investigated range (*σ*_layer_ < 100 nm), disorder induces only minor changes in *Q*_abs_ (~3%), while *Q*_scat_ is more strongly reduced, by up to 30%. Furthermore, under natural conditions, the illumination direction fluctuates widely with respect to the granal orientation, significantly differing from normal incidence. Figure 4b shows the absorption and scattering efficiencies as a function of the illumination angle *θ* for four morphologies with increasing *σ*_layer_. For all of them, the absorption efficiency is maximal at normal incidence *θ* = 0° and decreases monotonically to a minimum at *θ* = 90°. The decrease is maximal (~25%) for the largest investigated deviation *σ*_layer_ = 85 nm. As the standard deviation of the layer position increases, both the absorption and the scattering decrease, but the overall dependence on *θ* remains approximately unperturbed.

### Ensembles of grana

Approaching real chloroplasts more closely, we recall that, under many physiological conditions, the thylakoid membrane’s ultrastructure contains ensembles of grana separated by the stroma and stroma lamellae over a few hundred nanometres^2^. The results shown in the previous sections highlight that all grana scatter a significant amount of light, even for high chlorophyll concentrations up to *c*_protein_ = 300 mol L^−1^ (*k* = 0.05). Light scattering affects the optical response of neighboring grana through interference effects. Here we investigate coupling effects in representative ensembles of grana. In view of the computational costs, we only consider two-dimensional (2D) grana ensembles. This allows us to identify collective modes between grana and infer general trends as a basis for future studies of more complex ensembles.

**Fig. 5:**
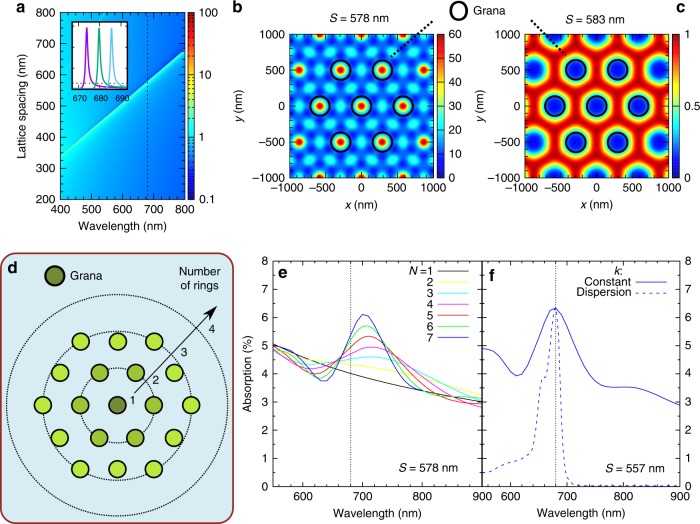
Upper row: an infinite ensemble of grana. **a** Absorption (mapped as color on a logarithmic scale) as a function of *S* and *λ* for *H* = 200 nm, AF = 20%, and *k* = 0.01. The inset shows spectra for *S* = 575, 578, and 583 nm and the spectrum of a slab with a dotted line. Spatial distribution of the light intensity in the plane at half-height for **b** *S* = 578 nm and **c** *S* = 583 nm. Bottom row: a finite ensemble of grana with *H* = 200 nm. **d** Schematic with a central granum and surrounding rings. **e** Absorption spectra (continuous lines) for ensembles with *S* = 578 nm and an increasing number of rings *N*, from 1–7. **f** Absorption spectra for ensembles with *N* = 7, *S* = 557 nm, and a constant extinction coefficient *k* = 0.01 (solid line), or *k* with spectral dispersion (dashed line)

The first geometry we address consists of an infinite hexagonal lattice of grana with (center-to-center) spacing *S*. Viewed from the direction of the incident wave, the grana fill only a fraction of the total area. For a fair comparison between different geometries, we keep the areal density fixed at AD = 20% so that the total absorbing volume is constant. Once *S* and AD are fixed, the granal diameter is uniquely determined: $D = S\sqrt {{\mathrm{AD}} \cdot 2\sqrt 3 /\pi }$. The absorption and scattering spectra in ensembles of dielectric nanoparticles are well known to be strongly dependent on geometric effects. Additionally, the absorption by the LHCs has its own wavelength dependence (given by the pigment composition and concentration), which affects the spectrum of *k*. Therefore, the absorption spectra of grana ensembles are a convolution of these two effects and will vary widely with species and illumination conditions. To simplify the interpretation of our calculations, we address only the role of nanoscale geometrical effects by setting *k* = 0.01 across the whole incident spectrum. Figure 5a shows a map of the absorption (in percent) as functions of the incident wavelength *λ* and *S*. The absorption level is low (<2.5%) except for a linear stripe approximately satisfying the relation *S* = *λ* × *n*_stroma_*/n*_granum_. Under this condition, the absorption rises to 37%. The inset shows representative spectra for *S* = 575, 578, and 581 nm. The value *S* = 578 nm was chosen because the ensemble features an absorption peak at *λ* ~ 680 nm. From these calculations, we conclude that an infinite ensemble of grana can selectively enhance the absorption of light by up to one order of magnitude at wavelengths controlled by the separation between grana. Moreover, from Fig. 5a and its inset, we notice that slight changes in the granal spacing (±3 nm) move the high-absorption condition off the P680 peak, as shown in the spectra with *S* = 575 and 581 nm.

To identify the origin of these low- and high-absorption states, we investigate the spatial distribution of light intensity at *λ* *=* 680 nm. Figure 5b, c show the cases for ensembles with *S* = 578 nm and *S* = 583 nm, respectively. In the latter case, the low-absorption state (Fig. 5c), the light intensity is negligible inside the grana and comparable to the incident radiation intensity (~1) in the surrounding stroma. In contrast, the intensity distribution in the high-absorption state (Fig. 5b) shows regions of high intensity inside the grana. Here the maximum local intensity is >50 times higher than the intensity of the incident radiation. These distributions reveal strong coupling between neighboring grana, in accordance with expectations arising from the previous analysis of light scattering. We conclude that the enhanced absorption results from efficient trapping of electromagnetic energy in the grana by means of collective light scattering. The constructive and destructive interference of the scattered field can sharpen the spectral response, giving rise to absorption peaks localized both in the spectrum and in space. In addition to the grana, the stroma lamellae, which bind PSI complexes and their antennas, also absorb light. To assess the effect of the lamellae on the ensemble optical response, we introduce an extinction coefficient to the stroma refractive index (in the range *k* = 0.01–0.1). However, this approach resulted in no additional peaks or other relevant features in the absorption spectra of the infinite ensemble. Therefore, for 2D ensembles, we conclude that the observed high-absorption state affects only the grana and not the stroma lamellae.

In reality, the thylakoid membrane is confined within a chloroplast (which in higher plants typically ranges in size from ~4 to 6 µm), and ensembles of grana have a finite size^2^. In the following, we model finite granal ensembles by preserving the unit cells and spacing but consider only a finite number of rings around the central granum, as sketched in Fig. 5d. Figure 5e shows the absorption spectrum of the central granum as the number of rings *N* increases. The black curve corresponds to the case with only a central granum and shows no significant spectral features besides decreased absorption at longer wavelengths. As the number of rings increases, a local absorption maximum appears, its spectral position slightly shifts, its amplitude increases, and its spectral bandwidth decreases. In general, the absorption maximum of a finite granal ensemble is redshifted with respect to that of an infinite ensemble with equal granal spacing *S*. For *N* = 7, we observe an absorption maximum of 6.1% at *λ* = 700 nm. This value is 63% (1.63 times) higher than the absorption of the local minimum at *λ* = 640 nm and 57% higher than the absorption efficiency of an isolated granum. To obtain an absorption peak exactly at *λ* = 680 nm, we can simply tune the granal spacing. This is shown in Fig. 5f, where the blue solid curve is the absorption spectrum for an ensemble with *S* = 557 nm and *N* = 7. Therefore, we conclude that a finite ensemble of grana provides high- and low-absorption states in an analogous way to the ideal case of an infinite ensemble. Three main differences are observed: (1) a smaller absorption difference between the high- and low-absorption states, (2) a redshifted absorption peak if the granal spacing is kept fixed, and (3) a larger spectral bandwidth. The spatial distribution of light in a finite ensemble (shown in Suppl. Fig. S4a) is very similar to that of an infinite one (Fig. 5b), confirming that the high-absorption state is due to light trapping inside the grana. It is fair to point out that the intensity associated with the grana in the outer rings (Suppl. Fig. S4b) is quite different, with a significantly reduced amplitude. However, in this study, we have not taken into account the outer chloroplast membrane nor the three-dimensional geometry of the granal ensembles, which might have significant effects. It is interesting to note that the spectra shown in Fig. 5e and the blue solid spectrum of Fig. 5f also have an absorption band extending to shorter wavelengths (*λ* ~ 550 nm), which has never been observed in experimental studies of chloroplasts. As mentioned previously in the text, all our calculations are performed by setting *k* = 0.01 across the whole incident spectrum to selectively address nanophotonic geometrical effects. However, the actual spectrum of granal ensembles in chloroplasts is the convolution of nanophotonic effects and the spectral dispersion of the LHCs. In Fig. 5f, we show the absorption spectrum (dashed line) of a granal ensemble with *S* = 557 nm and *N* = 7 by taking into account the full spectral dispersion of the extinction coefficient *k* (see Suppl. Info.), with a maximum *k* = 0.01 at *λ* = 680 nm. This spectrum only shows absorption peaks around *λ* = 680 nm and clearly lacks the absorption band at shorter wavelengths. A more qualitative comparison with the experimental absorption spectra of chloroplasts is premature at this point because of the variety of plant species, protein compositions, and light adaptation conditions, as well as the approximations we made to ensure computational feasibility.

**Fig. 6:**
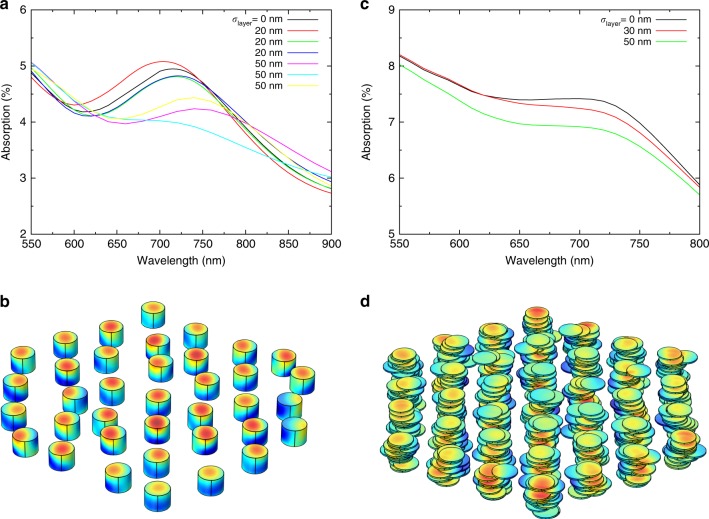
**a** Absorption spectra of ensembles of grana for different realizations of disorder with increasing standard deviation *σ*_granum_ of the positions of the grana (the spectrum of the ordered ensemble is shown with a black line) from 0 to 50 nm. **b** Light intensity distribution on the surfaces of the grana. **c** Absorption spectra of an ensemble of grana for three realizations of disorder in the position of the discoidal layers with *σ*_layer_ = 30 nm (red) and 50 nm (green). The black line shows the ordered ensemble with *σ*_layer_ = 0 nm. **d** Light intensity distribution on the surfaces of the granal layers

In real chloroplasts, granal ensembles are not ordered and display irregular spacings that vary with many factors, including species, acclimation state, and light conditions. Having shown that collective scattering modes exist for finite ordered ensembles, we now introduce disorder into the granal spacings. Specifically, we start from an ordered finite ensemble made of *N* rings and introduce lateral displacements in the absolute granal positions, with increasing standard deviation *σ*_granum_ from 0 (ordered) to 50 nm. Here we only address the nanophotonic effects and neglect the spectral dispersion of the extinction coefficient (i.e., we fix *k* = 0.01 across the spectral range of interest). Figure 6a shows the absorption spectra of a central granum in a disordered ensemble with *N* *=* *4*. For small (*σ*_granum_ = 20 nm) deviations, the absorption peak is preserved. In all realizations of disorder, resonance effects are clearly observed. Interestingly, some disordered cases feature even higher absorption than the ideal one. As the deviation increases (*σ*_granum_ = 50 nm), the absorption peak decreases and redshifts. The distribution of light intensity, shown in Fig. 6b, features hot spots similar to the ideal cases of the previous figures. In real chloroplasts, grana exhibit disorder in the stack as already addressed in Fig. 4 for the case of isolated grana. We study the effects of the lateral misalignment of the granal discoidal layers in finite ensembles (with *N* = 3 rings, due to computational costs). In this case, the resonances are preserved for small deviations (*σ*_layer_ up to 50 nm), and the peak gradually disappears if the deviation further increases, as shown in Fig. 6c. The related distribution of light intensity is shown in Fig. 6d. We infer that disorder in the granal spacing and the discoidal layer alignment within individual grana do not affect the collective modes giving rise to enhanced absorption if the level of disorder in terms of standard deviation is not higher than ~50 nm.

## Discussion

Plants have several well-known mechanisms to regulate light absorption across multiple scales under high light conditions^39^. In general, these mechanisms, such as the movements of leaves and chloroplasts, reduce the absorption efficiency through geometric means within the ray optics regime. Inside the chloroplast, we have shown that ray optics is insufficient to account for light propagation and absorption. Our results indicate that the ultrastructure of the thylakoid membrane in higher plants facilitates absorption regulation mechanisms based on nanophotonic geometric effects, which dominate at that scale.

Specifically, we considered grana at physiological conditions with a 70% volume fraction of proteins in the thylakoid membrane, which we represented as an effective medium with a homogeneous refractive index. By considering only changes in concentrations due to variation in the protein composition (i.e., the PSII:LHCII ratio), we found that the extinction coefficient spans a range from approximately 0.01 to 0.05. In addition to this nonlinear dependence on the protein composition, the chlorophyll concentration depends linearly on the protein volume fraction in the membrane. Values <70% can lead to an extinction coefficient of <0.01. As a consequence, our results indicated that the absorption efficiency of grana depends on protein composition and granal size, which vary with plant species and with adaptation to different illumination conditions. Our calculations further showed that a granum with a low chlorophyll concentration and large size scatters most of the incident light (*Q*_scat_ ∼ 6·*Q*_abs_ at *c*_protein_ ~ 60 mol L^−1^, for a granum of diameter *D* = 600 nm). Conversely, a granum with a high concentration and a small size is an efficient absorber that scatters only a small amount of light (*Q*_abs_ ∼ 3·*Q*_scat_ at *c*_protein_ ~ 300 mol L^−1^ for *D* = 200 nm). For intermediate cases found in grana under typical physiological conditions, light scattering and absorption have comparable magnitudes, and variations in the concentration and granal size can regulate this balance.

The presence of significant light scattering implies that interference effects between neighboring grana significantly affect the overall optical response of the chloroplast. We investigated ensembles of grana and found that collective scattering modes support states with increased absorption occurring in narrow spectral bands mostly controlled by the granal spacing. A representative 2D ordered finite ensemble of grana can increase absorption up to 2% with respect to the absorption at nonresonant wavelengths, and this value increases with the number of grana in the ensemble. We showed that this high-absorption state persists even in the presence of biological disorder, which is typically observed in structural studies^40^. A variation in the separation of grana, due to a change in their number or size, can disrupt this collective response and trigger a transition to a low-absorption state. We argue that this might be a strategy used by chloroplasts of higher plants to protect against very high light conditions.

Our calculations utilize the refractive index of grana as a parameter, quantified by means of effective medium theories, which assume that the medium is infinite. The effects of the finite size of the medium have been mostly overlooked in the literature. Recently, a few promising routes for development have been initiated for both random and periodic media^41–43^. In particular, ref. ^41^ provided a formula for random media that accounts for the finite size of the embedding (spherical) volume. The new formula approaches the traditional ones in the limit of large spheres. If the sphere is smaller than or comparable to the wavelength, the calculations show improvements in the estimation of the absorption over the traditional formulas. Effective medium theories for finite-size media are still in development, and it is reasonable to expect that, in the near future, there will be a deeper understanding and more numerical tools available. These results will allow us to further improve the accuracy of numerical studies of the nanophotonic effects in photosynthetic thylakoid membranes. We also showed that the spatial distribution of the absorbed light across the grana and their constituent discoidal layers is strongly nonuniform. This implies that the concentrations and compositions of protein complexes, both LHCs and PSs, may be adaptively and inhomogeneously distributed across the volumes of real grana. In future, it might be interesting to investigate the effects of this inhomogeneity on the nanophotonic functionalities we have found in our investigation.

Our work enables a deeper understanding of the optical response of chloroplasts in the full-wave optical regime, demonstrating for the first time functionalities intrinsic to thylakoid ultrastructure that are inexplicable using ray optics. Our results suggest the existence of an intermediate regime in photosynthetic systems in higher plants between macroscopic light transfer and excitation energy transfer at the protein level. We suggest that a better understanding of the nanophotonic transients driving LHCs at the nanoscale in vivo will require a comprehensive account of the full-wave optical effects characterized for the first time in the present study. Moreover, we speculate that extending our steady-state models to study nanophotonic transients in chloroplasts may offer new insights into the dynamics, quantum-coherent or otherwise, of excitation energy transfer.

As a consequence of the dynamic morphology of the thylakoid membrane and of a naturally varying light environment, the distributions of scattered and absorbed light continuously evolve across chloroplasts in higher plants. These light dynamics in the nanophotonic regime strongly affect the absorption of photons by the LHCs and therefore the photosynthetic productivity of the PSs. The ongoing study of adaptive self-organization in the thylakoid membrane may reveal new design principles for optical materials that can acclimate to different light conditions for applications in adaptive optics, imaging, and solar energy conversion. In particular, we envision the engineering of artificial photosynthetic systems with light management capabilities inspired by nature and based on nanophotonic principles. Moreover, incorporating the nanophotonic effects revealed here into the ever-growing understanding of complex composition–structure–function relationships in the chloroplast may enable new bioengineering approaches for plants and photosynthetic microorganisms. Microalgae, in particular, are now the focus of intense research for producing solar fuels and bioproducts, and it is known that so-called pseudograna form in the thylakoid membranes of some species^44^. This presents an opportunity for extending our work to ascertain whether nanophotonic effects might also be observed and exploited in microalgae.

In conclusion, in this work, we demonstrate that the morphological separation of the thylakoid membrane in higher plants facilitates nanophotonic effects based on changes in the concentrations of LHCs and on collective scattering between grana. These changes are known to occur in response to changing illumination conditions, providing the membrane with dynamic absorption regulation mechanisms. The intriguing adaptive self-organization of the thylakoid membrane in the nanophotonic regime not only unveils aspects of natural photosynthesis for the first time but will also inspire designs of new materials for adaptive optics, sensing, and solar energy. This opens new directions for the research and development of light-harvesting systems for bioengineered and bioinspired artificial photosynthetic systems with improved performance.

## Materials and methods

### Refractive index of the lumen and the stroma

The stroma and the lumen have aqueous compositions and contain a diverse set of proteins utilized for the dark- and light-dependent reactions of photosynthesis, respectively. The refractive indices of these two aqueous phases are close to that of water in the visible region (1.33–1.34) and only slightly increase with protein composition and pH. The protein composition has been reported to be on the order of ~20–40 mg mL^−1^^45^, implying a volume fraction of proteins of only a few percent. Therefore, in our calculations, we safely use a constant value of *n*_stroma_ = *n*_lumen_ = 1.35.

### Refractive index of the thylakoid membrane

The composition of the thylakoid membrane is highly heterogeneous, as shown in Fig S1a. In the following, we derive an effective index for the membrane *ñ*_thylakoid_ = *n*_thylakoid_ + *i·k*_thylakoid_ (Fig. S1b), where the tilde indicates a complex value.

A small fraction of the thylakoid consists of galactolipids. While their refractive index has not yet been addressed^46^, theoretical and experimental values for other types of lipids have been reported to be *n*_lipid_  ~ 1.55^47–49^. The remaining fraction (up to ~80% of the total volume) is occupied by functional proteins, such as PSII and LHCII. At the red absorption peak (wavelength *λ* = 680 nm), the light absorption by the protein phase is dominated by chlorophylls. Therefore, the extinction coefficient *k*_protein_ of the protein phase can be expressed as:

$$k_{{\mathrm{protein}}}\left( \lambda \right) = \frac{\lambda }{{4\pi }}\ln (10){\it{\epsilon }}\left( \lambda \right)c_{{\mathrm{protein}}}$$

where *λ* is the wavelength of the incident light, *ϵ* is the molar decadic absorption coefficient, and *c* is the molar chlorophyll concentration (in the protein phase). The molar absorption coefficient has been reported to be *∈* ~ 53000 M^−1^ cm^−1^ at *λ* = 680 nm^35^. The molar concentration *c*_protein_ varies widely with the composition of the protein phase; therefore, here, we identify three reference cases of composition: (A) only LHCIIs, (B) both PSIIs and LHCIIs (in a 1:1 ratio, i.e., assuming C_2_S_2_ supercomplex structure), and (C) only PSIIs. For each case, the chlorophyll concentration is:

$$c_{{\mathrm{protein}}} = \frac{N}{{VN_{\mathrm{A}}}}$$

where *N*_A_ is Avogadro’s number, *V* is the volume of the specific supercomplex, and *N* is the total number of chlorophylls in the supercomplex as extrapolated from the existing literature^50^. These values are listed in Table S1.

The spectrum (in arbitrary units) *k*_protein_(*λ*) can be obtained by measuring the absorption coefficient *α*_protein_(*λ*) experimentally (as shown in the inset of Fig. 2a) and by using the relation $k_{{\mathrm{protein}}}\left( \lambda \right) = \frac{\lambda }{{4\pi }}\alpha _{{\mathrm{protein}}}\left( \lambda \right)$. The absolute spectrum can be calibrated by using the value calculated at the wavelength *λ* = 680 nm. Having estimated the extinction coefficient, i.e., the imaginary part of the refractive index, we calculate its real part *n*_protein_ by using the well-known Kramers–Kronig relations and by assuming the short wavelength value *n*_protein_(*λ*→*0*) = *n*_lipid_. We show a complete spectrum for the complex refractive index *n*_protein_ in Fig. S2 for case B. The real part *n*_protein_(*λ*) shows only a small deviation (~5%) with respect to the short wavelength value *n*_protein_(*λ*→0). Lower chlorophyll concentrations will result in an even lower deviation; therefore, in the following, we simply assume *n*_protein_ = *n*_lipid_ = 1.55 across the whole spectrum of interest. This value is also in agreement with previous literature^37^.

Eventually, we calculate the effective index of the thylakoid membrane *ñ*_thylakoid_ (conceptually shown in Fig. S1b) by using Brugmann’s theory^51^, assuming a 30% volume fraction for the galactolipid phase and *f*_protein_ = 70% for the protein phase^36^. We show the value of the effective index at *λ* = 680 nm as a function of the chlorophyll concentration in Fig. 2a of the main text. All the relevant values for the three reference cases of protein composition are listed in Table S1.

### Refractive index of the granum as a stratified medium

We use the theory of periodic stratified media to calculate the effective refractive index *ñ* = *n* *+* *i·k* of a discoidal layer of grana (where the *strata* are two lipid bilayer membranes, the lumen and the stroma, as shown in Fig. 1c, d of the main text)^52^. This procedure results in an anisotropic index. However, the difference between the ordinary and extraordinary values is negligible, and in the following, we calculate an isotropic value *ñ* as the average of the two. Again, *ñ* depends on a number of parameters, including the protein composition, the thickness of the strata, and the wavelength. For the calculations in the main text, we fix the thicknesses of the thylakoid, lumen, and stroma strata at *t*_thylakoid_ = 3 nm, *t*_lumen_ = 7.5 nm, and *t*_stroma_ = 1.8 nm, as found in the literature^16^. With these values, the extinction coefficients for the three reference cases of protein composition are listed in Table S1.

In conclusion, our analysis of the effective refractive index in the grana allows us to identify a range of reasonable values for the effective extinction coefficient *k* that we can utilize in the calculations presented in the main manuscript. For the real index *n*, we proceed by fixing its value at *n* = *n*_lipid_ = 1.55 for two main reasons: first, to maintain consistency with the existing literature; second, because it turns out that the optical properties calculated in the manuscript only slightly depend on the exact value of *n*. The low optical contrast with the aqueous phases results in a value of *n* that is only ~8% lower than *n*_grana_.

It is also known that the lumen can expand in reaction to changing illumination and chemical conditions. To assess the effect of this change in volume on the refractive index, in the following, we estimate *ñ* for two values of lumen thickness: *t*_lumen_ = 4.7 and 8.8 nm, as reported for the cases of dark- and light-adapted thylakoids, respectively^15^. We fix the thickness of each of the two lipid bilayers at 4 and 3.6 nm for the stroma, as reported in the same reference. In Table S2, we summarize six values derived for *ñ*, depending on the composition of the protein phase and the lumen thickness at *λ* = 680 nm. We conclude that the extinction coefficient *k* changes more dramatically with protein composition than with lumen thickness. Therefore, we can safely neglect changes in the refractive index due to the expansion of the lumen.

An important avenue for extending our work is to account for the psi-type circular dichroism known to occur in grana due to the chiral structure at multiple scales, primarily the chiral order of protein arrays at the scale of an overall granum^20^. Its physical origin has previously been explained using an approximate analytical theory of interactions between light and large molecular aggregates with chiral structure^53^. In our study, we solve the full-wave Maxwell equations, and the effects of the chiral structure may be included by considering anisotropy in the effective refractive index of the thylakoid.

## Supplementary information

Supplementary Information

## References

[1] Ringsmuth AK, Landsberg MJ, Hankamer B (2016). Can photosynthesis enable a global transition from fossil fuels to solar fuels, to mitigate climate change and fuel-supply limitations?. Renew. Sustain Energy Rev..

[2] Dekker JP, Boekema EJ (2005). Supramolecular organization of thylakoid membrane proteins in green plants. Biochim. Biophys. Acta Bioenerg..

[3] Uwada T, Huang LT, Hee PY, Usman A, Masuhara H (2017). Size-dependent optical properties of grana inside chloroplast of plant cells. J. Phys. Chem. B.

[4] Anderson JM, Chow WS, De Las, Rivas J (2008). Dynamic flexibility in the structure and function of photosystem II in higher plant thylakoid membranes: the grana enigma. Photosynth. Res..

[5] Mullineaux CW (2005). Function and evolution of grana. Trends Plant Sci..

[6] Croce R, van Amerongen H (2014). Natural strategies for photosynthetic light harvesting. Nat. Chem. Biol..

[7] Anderson JM (1986). Photoregulation of the composition, function, and structure of thylakoid membranes. Annu. Rev. Plant Physiol..

[8] Anderson JM, Chow WS, Goodchild DJ (1988). Thylakoid membrane organisation in sun/shade acclimation. Aust. J. Plant Physiol..

[9] Wientjes E, Van Amerongen H, Croce R (2013). LHCII is an antenna of both photosystems after long-term acclimation. Biochim. Biophys. Acta Bioenerg..

[10] Kouřil R, Wientjes E, Bultema JB, Croce R, Boekema EJ (2013). High-light vs. low-light: effect of light acclimation on photosystem II composition and organization in *Arabidopsis thaliana*. Biochim. Biophys. Acta Bioenerg..

[11] Rochaix JD (2014). Regulation and dynamics of the light-harvesting system. Annu. Rev. Plant Biol..

[12] Goldschmidt-Clermont M, Bassi R (2015). Sharing light between two photosystems: mechanism of state transitions. Curr. Opin. Plant Biol..

[13] Anderson JM, Horton P, Kim EH, Chow WS (2012). Towards elucidation of dynamic structural changes of plant thylakoid architecture. Philos. Trans. R. Soc. B Biol. Sci..

[14] Wood WHJ (2018). Dynamic thylakoid stacking regulates the balance between linear and cyclic photosynthetic electron transfer. Nat. Plants.

[15] Kirchhoff H (2011). Dynamic control of protein diffusion within the granal thylakoid lumen. Proc. Natl. Acad. Sci. USA.

[16] Daum B, Nicastro D, Austin J, McIntosh JR, Kühlbrandt W (2010). Arrangement of photosystem II and ATP synthase in chloroplast membranes of spinach and pea. Plant Cell.

[17] Fristedt R (2009). Phosphorylation of photosystem II controls functional macroscopic folding of photosynthetic membranes in *Arabidopsis*. Plant Cell.

[18] Khatoon M (2009). Quality control of photosystem II: thylakoid unstacking is necessary to avoid further damage to the D1 protein and to facilitate D1 degradation under light stress in spinach thylakoids. J. Biol. Chem..

[19] Herbstová M, Tietz S, Kinzel C, Turkina MV, Kirchhoff H (2012). Architectural switch in plant photosynthetic membranes induced by light stress. Proc. Natl. Acad. Sci. USA.

[20] Garab G, Mustárdy L (1999). Role of LHCII-containing macrodomains in the structure, function and dynamics of grana. Aust. J. Plant Physiol..

[21] Janik E (2013). Molecular architecture of plant thylakoids under physiological and light stress conditions: a study of lipid-light-harvesting complex II model membranes. Plant Cell.

[22] Chuartzman SG (2008). Thylakoid membrane remodeling during state transitions in *Arabidopsis*. Plant Cell Online.

[23] Liberton M, Howard Berg R, Heuser J, Roth R, Pakrasi HB (2006). Ultrastructure of the membrane systems in the unicellular cyanobacterium *Synechocystis* sp. strain PCC 6803. Protoplasma.

[24] Mullineaux CW (2014). Electron transport and light-harvesting switches in cyanobacteria. Front. Plant Sci..

[25] Watanabe M, Ikeuchi M (2013). Phycobilisome: architecture of a light-harvesting supercomplex. Photosynth. Res..

[26] Ranjbar Choubeh R, Wientjes E, Struik PC, Kirilovsky D, van Amerongen H (2018). State transitions in the cyanobacterium *Synechococcus elongatus* 7942 involve reversible quenching of the photosystem II core. Biochim. Biophys. Acta Bioenerg..

[27] Durnford DG (1999). A phylogenetic assessment of the eukaryotic light-harvesting antenna proteins, with implications for plastid evolution. J. Mol. Evol..

[28] Engel BD (2015). Native architecture of the *chlamydomonas* chloroplast revealed by in situ cryo-electron tomography. eLife.

[29] Terashima I, Hanba YT, Tholen D, Niinemets Uuml (2011). Leaf functional anatomy in relation to photosynthesis. Plant Physiol..

[30] Wada M (2013). Chloroplast movement. Plant Sci..

[31] Capretti A, Lesage A, Gregorkiewicz T (2017). Integrating quantum dots and dielectric Mie resonators: a hierarchical metamaterial inheriting the best of both. ACS Photonics.

[32] Margalit O, Sarafis V, Zalevsky Z (2010). The effect of grana and inter-grana components of chloroplasts on green light transmission: a preliminary study. Optik.

[33] Jacobs M (2016). Photonic multilayer structure of *Begonia* chloroplasts enhances photosynthetic efficiency. Nat Plants.

[34] Vignolini S, Moyroud E, Glover BJ, Steiner U (2013). Analysing photonic structures in plants. J. R. Soc. Interface.

[35] Paillotin G, Dobek A, Breton J, Leibl W, Trissl HW (1993). Why does the light-gradient photovoltage from photosynthetic organelles show a wavelength-dependent polarity?. Biophys. J..

[36] Kirchhoff H (2008). Molecular crowding and order in photosynthetic membranes. Trends Plant Sci..

[37] Cinque G, Croce R, Holzwarth A, Bassi R (2000). Energy transfer among CP29 chlorophylls: Calculated förster rates and experimental transient absorption at room temperature. Biophys. J..

[38] Ruban AV, Johnson MP, Duffy CDP (2012). The photoprotective molecular switch in the photosystem II antenna. Biochim. Biophys. Acta Bioenerg..

[39] Taiz, L. & Zeiger, E. *Plant Physiology* (Sinauer Associates, Sunderland, 2010).

[40] Nevo, R. et al. in *Lipids in Photosynthesis: Essential and Regulatory Functions* (eds Wada, H. & Murata, N.) 295–328 (Springer, Dordrecht, 2009).

[41] Guérin CA, Mallet P, Sentenac A (2006). Effective-medium theory for finite-size aggregates. J. Opt. Soc. Am. A..

[42] Markel VA, Schotland JC (2012). Homogenization of Maxwell’s equations in periodic composites: Boundary effects and dispersion relations. Phys. Rev. E.

[43] Tsukerman I (2017). Classical and non-classical effective medium theories: new perspectives. Phys. Lett. A.

[44] Goodenough UW, Levine RP (1969). Chloroplast ultrastructure in mutant strains of *Chlamydomonas reinhardi* lacking components of the photosynthetic apparatus. Plant Physiol..

[45] Kieselbach T, Hagman Aring, Andersson B, Schröder WP (1998). The thylakoid lumen of chloroplasts. Isolation and characterization. J. Biol. Chem..

[46] Van Eerden FJ, De Jong DH, De Vries AH, Wassenaar TA, Marrink SJ (2015). Characterization of thylakoid lipid membranes from cyanobacteria and higher plants by molecular dynamics simulations. Biochim. Biophys. Acta Biomembr..

[47] Ohki S (1968). Dielectric constant and refractive index of lipid bilayers. J. Theor. Biol..

[48] Den Engelsen D (1976). Optical anisotropy in ordered systems of lipids. Surf. Sci..

[49] Huang W, Levitt DG (1977). Theoretical calculation of the dielectric constant of a bilayer membrane. Biophys. J..

[50] Wei XP (2016). Structure of spinach photosystem II–LHCII supercomplex at 3.2 Å resolution. Nature.

[51] Milton GW (2002). The Theory of Composites. Cambridge Monographs on Applied and Computational Mathematics.

[52] Yariv A, Yeh P (1977). Electromagnetic propagation in periodic stratified media. II. Birefringence, phase matching, and x-ray lasers. J. Opt. Soc. Am..

[53] Keller D, Bustamante C (1986). Theory of the interaction of light with large inhomogeneous molecular aggregates. II. Psi-type circular dichroism. J. Chem. Phys..

