# Supplementary material for: Nanophotonics of higher-plant photosynthetic membranes
Source: Light Sci Appl. 2019 Jan 9;8:5. doi: 10.1038/s41377-018-0116-8 (PMC6325066; doi:10.1038/s41377-018-0116-8)
Supplement: Supplementary file 1 — Supplementary Information [file 41377_2018_116_MOESM1_ESM.pdf]

Supplementary information
Nanophotonics of higher-plant photosynthetic membranes

A. Capretti,[1*] A.K. Ringsmuth,[2†] J. van Velzen,[1,2] A. Rosnik,[3] R. Croce,[2] and T. Gregorkiewicz[1]

[1] Institute of Physics, University of Amsterdam, Netherlands
[2] Dep. Physics and Astronomy, VU University Amsterdam, Netherlands
[3] College of Chemistry, University of California, Berkeley, USA

[*] E-mail: a.capretti@uva.nl.
[†] Current address: Stockholm Resilience Centre, Stockholm University, Sweden.

Refractive indices

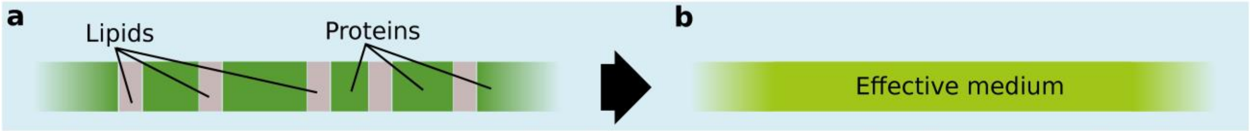

Figure S1. a) The thylakoid membrane is a heterogeneous medium made of a lipid phase and a protein phase. b) Using an effective medium theory, we model the thylakoid as a homogeneous medium having refractive index $\tilde{n}_{thylakoid}$.

Protein composition	Volume (nm^3)	N. of chl.	Molar conc. of chl. (10^{-3} $mol\ L^{-1}$)	Ext. coeff. $k_{protein}$	Ext. coeff. $k_{thylakoid}$	Ext. coeff. k
(A) LHCII	242	42	288.2	0.190	0.133	0.048
(B) PSII-LHCII	4004	156	85.4	0.056	0.039	0.014
(C) PSII	1144	35	50.8	0.034	0.023	0.009

Table S1. Parameters used to estimate $k_{protein}$, $k_{thylakoid}$ and k at $\lambda=680\ nm$, for three reference cases of the protein composition: (A) only LHCIIs, (B) both PSIIs and LHCIIs (in a 1:1 ratio) and (C) only PSIIs.

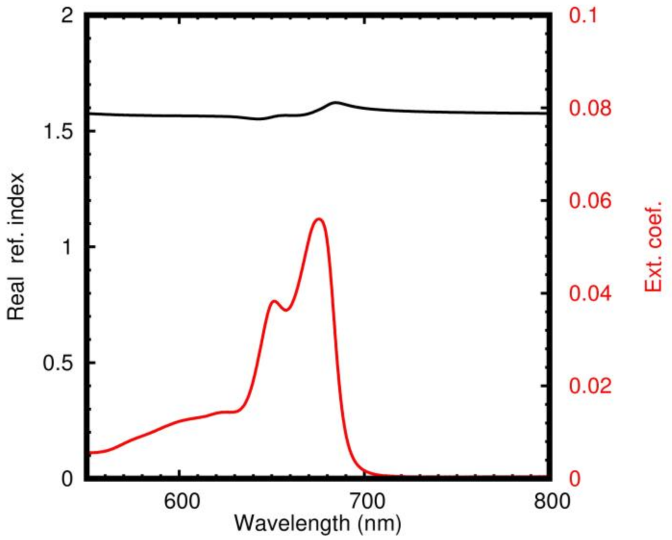

Figure S2. Real (left axis) and imaginary (right axis) parts of the refractive index $\tilde{n}_{protein}$ for the protein phase in the composition case B (protein phase occupied by both PSIIs and LHCIIs in a *1:1* ratio).

Protein composition	dark-adapted	light-adapted
(A) LHCII	*0.061*	*0.048*
(B) PSII-LHCII	*0.018*	*0.014*
(C) PSII	*0.011*	*0.009*

Table S2. Extinction coefficient at $\lambda = 680\ nm$ of the discoidal layer of the grana for dark- and light-adapted thylakoids, and for three reference cases of the protein composition: (A) only LHCIIs, (B) both PSIIs and LHCIIs (in a *1:1* ratio) and (C) only PSIIs.

Definitions of optical quantities and numerical methods

Individual grana. The absorption Q_{abs} and scattering Q_{scat} cross-sections of an individual granum illuminated by a plane-wave are defined as:[1]

$$Q_{abs} = \frac{k_0}{A} imag \iiint_V \varepsilon_2 \frac{|\boldsymbol{E}|^2}{|\boldsymbol{E}_0|^2} dV \qquad ; \qquad Q_{scat} = \frac{k_0}{A}\ real \iint_S \frac{\boldsymbol{E}_S \times \boldsymbol{H}_S^*}{\boldsymbol{E}_0 \times \boldsymbol{H}_0^*}\ dS$$

where V is the volume of the granum, S its surface, A is the geometrical cross-section at normal incidence (the area of the cylinder base), ε_2 its imaginary permittivity, k_0 the wavenumber of the incident plane-wave, $(\boldsymbol{E}_0, \boldsymbol{H}_0)$ is the incident electromagnetic field, and $(\boldsymbol{E}_s, \boldsymbol{H}_s)$ is the scattered electromagnetic field. In this work we use the transition matrix method, also known as null-field, to calculate the full-wave solution of Maxwell Equations and the scattering and absorption cross-sections.[2] In this method, the expansion coefficients for the scattered field are retrieved by combining the null-field equation with the boundary conditions. We also use a finite element method (FEM) to calculate the cross-sections for the cases of stratified granum and of grana made of displaced disks, and for the spatial distribution of light.

Ensembles of grana. We use the rigorous coupled wave approach (RCWA), also known as Fourier modal method, to calculate the absorption spectra of the 2D infinite ensembles of grana.[3] For finite ensembles we use FEM as for the case of individual grana.

Light intensity distribution averaged across grana with lateral displacement of their discoidal layers

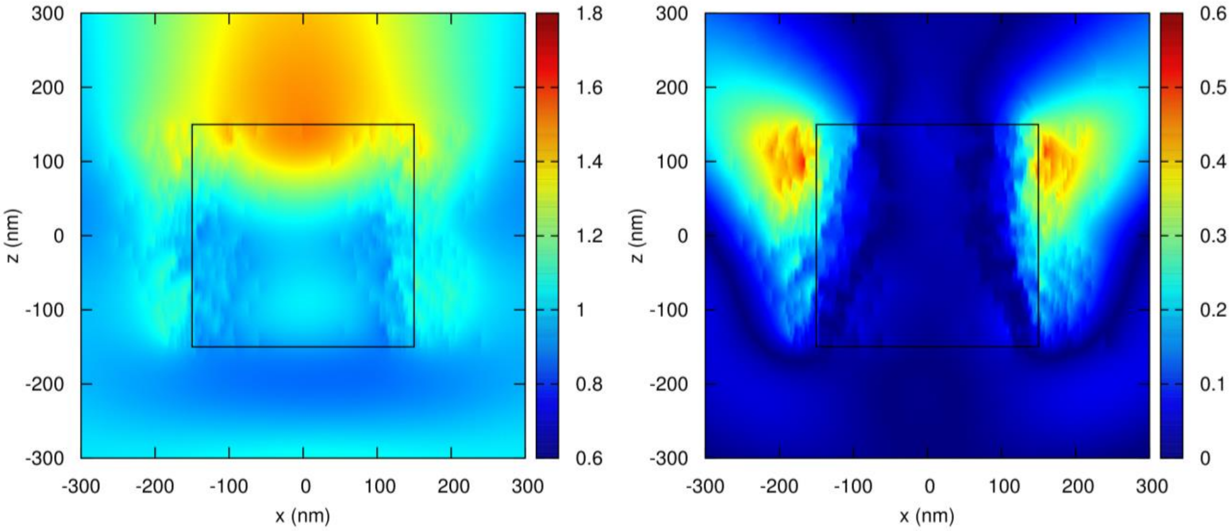

Figure S3. a) Spatial distributions of light intensity calculated as an average across ten realizations of grana made of laterally-displaced discoidal layers of thickness *15.3 nm*. Each layer is homogeneous with $k=0.01$. The lateral displacements with respect to the *x*- and *y-axes* are sampled from Gaussian distributions with $\sigma_{layer} = 40\ nm$. Incident light is a plane-wave propagating along the *z*-axis from above with $\lambda=680\ nm$, linearly polarized along the *x*-axis and with unitary intensity. b) Difference between the light distribution of the cylindrical granum of Fig. 3b of the main article, and the distribution of panel a) of the present figure. In both panels, the rectangle indicates the side view of the cylindrical granum of Fig. 3b of the main article

Light intensity distribution for finite ensembles of grana

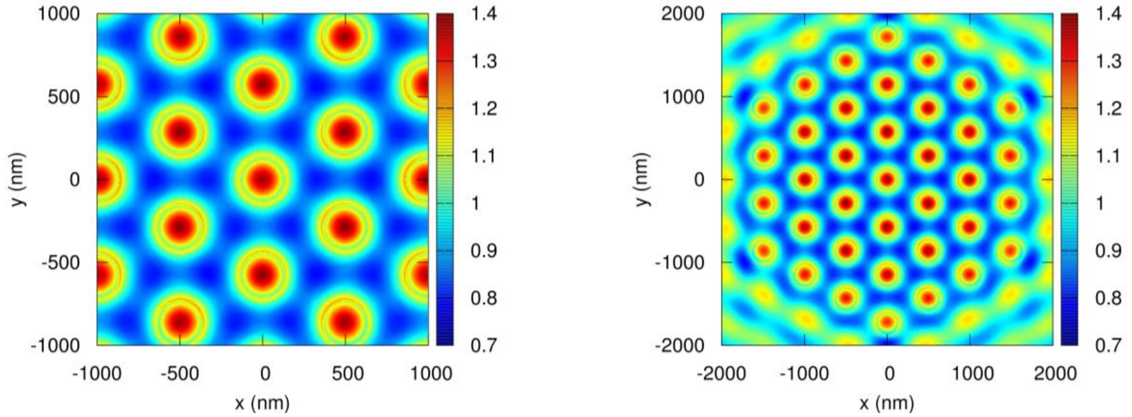

Figure S4. a) Hexagonal ensemble composed of 4 rings of grana with $H=200nm$ and $S=578nm$: spatial distribution of light intensity in the plane at half-height of the grana at $\lambda=680nm$. b) Same as panel a) but in a larger spatial range, in order to show the external grana of the ensemble.

References

(1) Bohren, C. F.; Huffman, D. R. *Absorption and Scattering of Light by Small Particles*; 1998; Vol. 16.

(2) Doicu, A.; Wriedt, T.; Eremin, Y. A. *Light Scattering by Systems of Particles*; 2006; Vol. 124.

(3) Liu, V.; Fan, S. S 4: A Free Electromagnetic Solver for Layered Periodic Structures. *Comput. Phys. Commun.* **2012**, *183*, 2233–2244.
